# Anti-hyperglycemic effects of *Cissus quadrangularis* extract via regulation of gluconeogenesis in type 2 diabetic *db/db* mice

**DOI:** 10.3389/fphar.2024.1415670

**Published:** 2024-07-10

**Authors:** Jeong-Won Kim, Ji-Soo Jeong, Jin-Hwa Kim, Eun-Hye Chung, Chang-Yeop Kim, Dong-Ryung Lee, Bong-Keun Choi, Jong-Hwan Lim, Je-Won Ko, Tae-Won Kim

**Affiliations:** ^1^ College of Veterinary Medicine (BK21 FOUR Program), Chungnam National University, Daejeon, Republic of Korea; ^2^ Research Institute, NUON Co., Ltd., Seongnam, Republic of Korea; ^3^ EveryH Co., Ltd., Seoul, Republic of Korea

**Keywords:** anti-hyperglycemic effect, *Cissus quadrangularis*, gluconeogenesis, lipogenesis, oxidative stress, type 2 diabetes mellitus

## Abstract

**Introduction:** *Cissus quadrangularis* is a vining plant widely used as a traditional herbal remedy for various ailments. In this study, the therapeutic effects of *C. quadrangularis* extract (CQR-300) on type 2 diabetes mellitus (T2DM) were investigated in a leptin receptor-mutated *db/db* mouse model.

**Methods:** CQR-300 was orally administered to *db/db* mice (n = 6/group) at different doses (50, 100, and 200 mg/kg) for 8 weeks. Blood glucose levels and oral glucose tolerance were assessed using the AccuCheck glucometer. Enzyme-linked immunosorbent assay was performed to evaluate insulin and hemoglobin A1c (HbA1c) levels in the blood of *db/db* mice. Liver and pancreatic tissues from *db/db* mice were examined by hematoxylin and eosin (H&E) and immunohistochemical staining. The protein levels of gluconeogenesis-, lipogenesis-, and oxidative stress-related factors were evaluated using western blotting.

**Results and discussion:** CQR-300 treatment effectively reduced body weight, blood glucose, and insulin levels. HbA1c levels were increased by leptin receptor mutation. Additionally, in the oral glucose tolerance tests, the CQR-300 treated group had a faster blood glucose recovery rate than the *db/db* group. H&E and Oil red-O staining of the liver showed decreased lipid accumulation in the CQR-300 treated group than the *db/db* group. Western blot analysis confirmed that CQR-300 effectively inhibited gluconeogenesis, lipogenesis, and oxidative stress-related factors. Our findings suggest that CQR-300 has the potential to be used as a T2DM supplement.

## 1 Introduction

Obesity has reached epidemic proportions and has become a major public health and economic issue, characterized by an imbalance between energy intake and expenditure (Jaison et al., 2024). Obesity triggers not only cardiovascular diseases but also various metabolic comorbidities, including insulin resistance, type 2 diabetes mellitus (T2DM), and liver diseases, which mutually contribute to the exacerbation of comorbidities (Godoy-Matos et al., 2020). Fat accumulation in obesity leads to insulin resistance, which increases insulin secretion. Subsequently, the pancreatic insulin secretion capacity is impaired, ultimately leading to the development of diabetes (Ahmed et al., 2021). Recently, there has been increasing recognition of the mutual impact of obesity on disease progression and the potential for significant improvement in diabetes through weight loss, which is regarded as a primary treatment goal rather than blood sugar control alone (Lingvay et al., 2022).

*Cissus quadrangularis*, a vining plant predominantly native to India, has gained extensive recognition as a herbal remedy with a broad range of applications, including pain relief, fever reduction, anti-inflammatory effects, bone fracture healing, tissue repair, and weight loss (Stohs and Ray, 2013). Recently, anti-obesity has become a prominent indication for issue-originated products, and numerous studies have been conducted using well-controlled human trials and obesity models, providing substantial support for its clinical application (Sawangjit et al., 2017; Nash et al., 2019). Previous studies have indicated the biological effect of *C. quadrangularis* in reducing body fat through the regulation of fatty acid synthesis in diet-induced obesity mice models (Lee et al., 2016). Moreover, the stems of *C. quadrangularis* have been shown to alleviate insulin resistance, oxidative injury, and fatty liver disease in fructose-fed rats (Chidambaram and Carani Venkatraman, 2010). Furthermore, an anti-steatohepatitis effect has been reported in the *C. quadrangularis*-treated T2DM rat model (Syed et al., 2021). However, the mechanism underlying its anti-hyperglycemic effect remains unclear, and the effect of *C. quadrangularis* on T2DM remains unknown.

The present study was to evaluate the impact of CQR-300 (0.3% of quercetin) on blood glucose levels in *db/db* mice and its potential therapeutic efficacy related to the exacerbation of diabetes.

## 2 Materials and methods

### 2.1 CQR-300 preparation and quercetin analysis

*C. quadrangularis* extract (CQR-300) powder was purchased from Gateway Health Alliance (Fairfield, CA, Unites States). The stems and leaves of *C. quadrangularis* Linn. were used to extract the powder, and India was the place of production. In brief, the raw material (150 kg) was soaked in pure water (525 L), boiled, and concentrated at 90°C under a vacuum (400–800 mbars) for 3 h. Then, the supernatant was spray-dried and sieved. The extraction yield was 25 kg (16.7%). CQR-300 was dissolved in saline for *in vivo* studies.

High-performance liquid chromatography (HPLC) analysis was conducted using an ultraviolet (UV) detector (Agilent Technologies, Santa Clara, CA). The standard chemicals (quercetin, ≥ 95% purity; kaempferol, ≥ 97% purity) were obtained from Sigma-Aldrich (St. Louis, MO). The analysis was performed using a C18 column (2.1 mm × 150 mm, 3.5 μm; Agilent) at 40°C. The mobile phase was consisted of 0.1% formic acid (in distilled water) (A) and acetonitrile (B). A gradient solvent program was run as follows: 0–1 min (97% A), 1–13 min (97%–50%, A), 13–20 min (50%–0%), 20–27 min (0%–97%). The wavelength of detection was 205 nm and the injection volume was 5 μL. The flow rate was 0.25 mL/min.

### 2.2 Animals and environmental conditions

All experimental procedures were approved by the Institutional Animal Care and Use Committee of Chungnam National University (approval no. 202310A-CNU-174) and were performed following the guidelines for animal experiments at Chungnam National University. Male *db/db* mice (6 weeks old, body weight 31–32 g) were purchased from Jackson Laboratory (Bar Harbor, ME, Unites States). Mice were housed in standard conditions (humidity 50% ± 10%, temperature 23°C ± 3°C, 13–18 air changes/h, and 12 h light/dark cycles). Commercial rodent chow and tap water were provided *ad libitum*. After an acclimatization period of 14 days, the mice were allocated to five groups (n = 6) according to their body weight: (1) wild type C57BL/6 mouse with phosphate-buffered saline (PBS) (normal control [NC] group); (2) *db/db* mouse with PBS (*db/db* group); (3), (4), (5) *db/db* mouse with 50, 100, and 200 mg/kg of CQR-300 (CQR-300 50 mg/kg, CQR-300 100 mg/kg, and CQR-300 200 mg/kg group). The mice were randomly divided into five groups, and the body weight difference between groups was within 1 g. CQR-300 was dissolved in PBS with 10 mL/kg and administered daily via oral gavage for 8 weeks. According to Kothari et al. (2011), the NOAEL (no-observed-adverse-effect level) when *C. quadrangularis* extract was orally administered to rats for 90 days was evaluated at 2,500 mg/kgbw/day. Also, Bafna et al. (2021) reported that LD50 was over 3,000 mg/kgbw/day when orally administered to rats for 90 days. Based on these previous studies, the concentration was set at 50, 100, and 200 mg/kgbw/day. The body weight and food and water intake of the mice were measured weekly.

### 2.3 Oral glucose tolerance test

Oral glucose tolerance test (OGTT) was performed on day 56 after 15 h of fasting. The mice received an oral glucose load (1.5 mg/kg). Blood glucose levels were measured in the tail vein blood at fasting and 30, 60, and 120 min of glucose administration using an Accu-Check glucometer (Roche Diagnostics, Mannheim, Germany).

### 2.4 Enzyme-linked immunosorbent assay

Blood was collected from the inferior vena cava 56 days after CQR-300 treatment. The levels of hemoglobin A1c (HbA1c) and insulin in the blood were measured using enzyme-linked immunosorbent assay (ELISA) kits (Crystal Chemical Inc., Belvidere, IL, Unites States), according to the manufacturer’s protocol. Absorbance was measured at 700 and 450 nm using a microplate reader (iMarkTM; Bio-Rad Laboratories, Hercules, CA, Unites States).

### 2.5 Histopathological/immunohistochemical examination

The liver and pancreatic tissues were processed as previously described (Kim et al., 2023). The tissues were stained with hematoxylin and eosin (H&E; BBC Biochemical, Mount Vemon, WA, Unites States). Insulin and glucagon proteins in the pancreas were stained using an immunohistochemistry (IHC) kit (Abcam, Cambridge, Unites States) following the manufacturer’s instructions. Primary antibodies against insulin (1:1000; Abcam) and glucagon (1:1000; Abcam) were used for IHC staining. The brown-stained cells were positive for insulin and glucagon. Quantitative analyses were conducted with a light microscope in a blinded manner (Leica Microsystems, Wetzlar, Germany) at 10 × and 20 × objective lenses using IMT i-Solution software (Vancouver, North Road Burnaby, Canada).

### 2.6 Oil red O staining

Frozen liver tissues were sectioned at 6 µm and the slides were fixed in 4% paraformaldehyde for 30 min. Lipid accumulation in the liver was evaluated using an Oil Red O staining assay according to the manufacturer’s protocol (Abcam). Quantitative analyses were conducted with a light microscope in a blinded manner (Leica Microsystems) at 10 × and 20 × objective lenses using IMT i-Solution software.

### 2.7 Determination of antioxidant markers in liver tissue

Malondialdehyde (MDA), glutathione peroxidase (GPX), and superoxide dismutase (SOD) were detected in the liver tissues of mice from all five groups. MDA, GPX, and SOD assay kits were purchased from Cayman Chemicals (Ann Arbor, MI, Unites States) and used in strict accordance with the manufacturer’s protocols. The absorbance (450 nm) was measured using a microplate reader (Bio-Rad Laboratories).

### 2.8 Western blot

The frozen liver tissues were homogenized with tissue lysis/extraction kit (Sigma-Aldrich) containing a protease/phosphatase inhibitor cocktail (Sigma-Aldrich) and centrifuged at 16,000 × *g* for 10 min at 4°C to isolate the proteins in the lysate. Immunoblotting was performed as described previously (Kim et al., 2023) to examine the levels of proteins related to gluconeogenesis, lipogenesis, and oxidative stress.

For gluconeogenesis-related factors, the expression levels of insulin receptor substrate 1 (IRS-1), AMP-activated protein kinase (AMPK), protein kinase B (AKT), phosphoinositide 3-kinases (PI3K), glycogen synthase kinase-3β (GSK-3β), forkhead box O1 (FOXO1), glucose 6-phosphatase (G6pase), and phosphoenolpyruvate carboxykinase (PEPCK) were evaluated. To assess the expression of lipogenesis-related factors, acetyl-CoA carboxylase (ACC), sterol regulatory element-binding protein 1 (SREBP-1), and fatty acid synthetase (FAS) were analyzed. To evaluate the oxidative stress-related factors, the expression levels of NADPH oxidase 4 (NOX4), catalase (CAT), GPX, glutathione reductase (GR), SOD-1, and SOD-2 were measured. The distributors and dilutions of the antibodies are shown in Supplementary Table S1. Each protein band was photographed using a ChemiDoc (Bio-Rad Laboratories). Protein levels were normalized to β-actin.

### 2.9 Statistical analysis

The results were expressed as mean ± standard deviation. To calculate statistical significance, one-way analysis of variance followed by *post hoc* Tukey’s honest significant difference test was used. All calculations were performed using the GraphPad InStat software (version 3.0; GraphPad Software, Inc., CA, Unites States). Significance was indicated by a *p*-value less than 0.05 or 0.01.

## 3 Results

### 3.1 HPLC analysis of CQE-300

**FIGURE 1:**
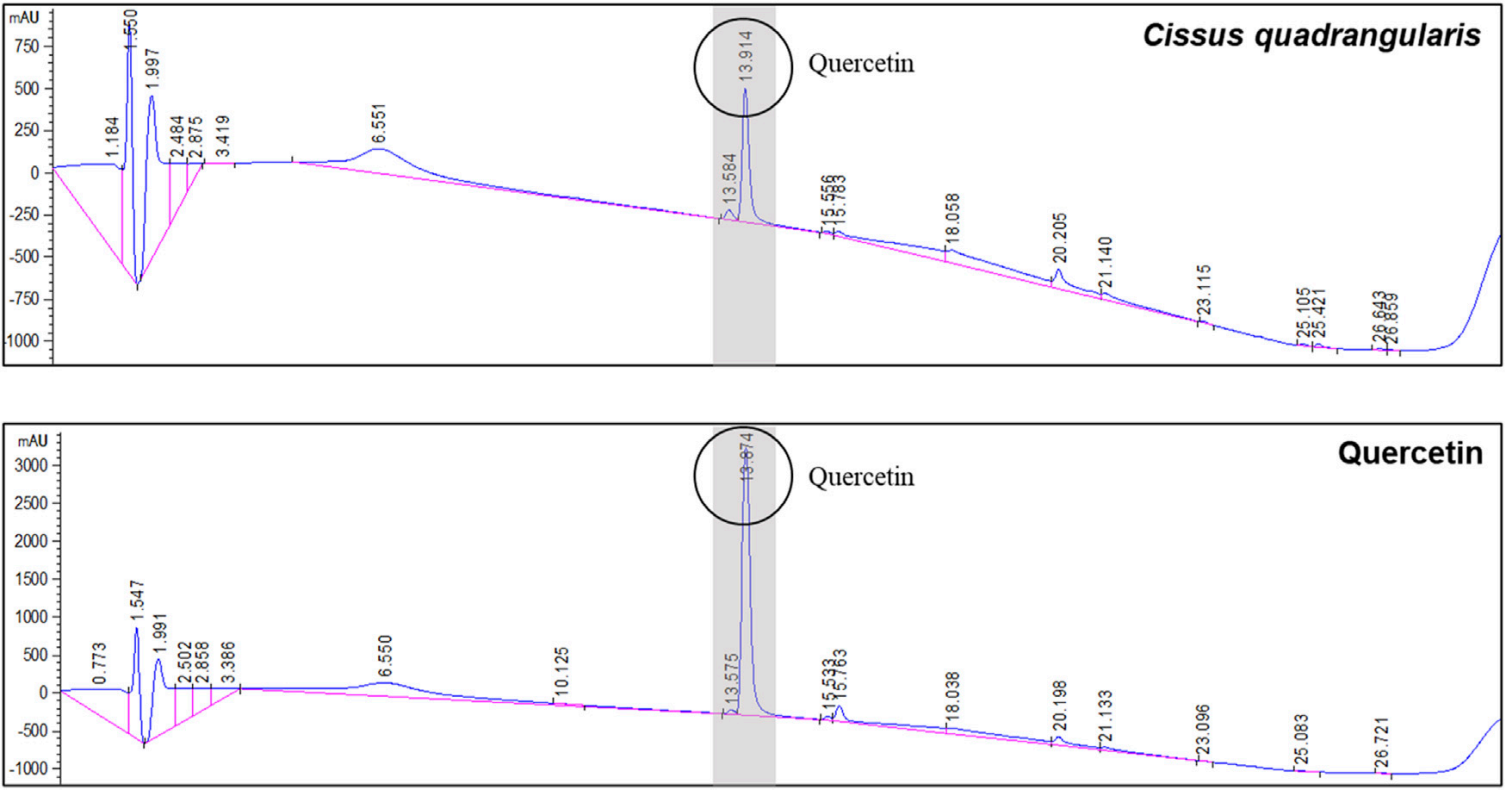
CQE-300 contains 1.15% quercetin. HPLC analysis of CQE-300 using a UV detector for quantitative analysis of quercetin. Quercetin was detected after 13.914 min at 205 nm. CQR-300: *Cissus quadrangularis* extract. HPLC, high performance liquid chromatography, UV, ultra-violet.

CQE-300 was analyzed by quercetin content using HPLC-UV (Figure 1). It was analyzed at 205 nm. The quercetin contents in CQE-300 were identified by its retention time with quercetin standard. The retention time for quercetin was 13.914 min, and the quercetin content in CQE-300 was 1.15%. The catechin and kaempferol content were below the detection limit.

### 3.2 Effect of CQR-300 on body weight, and insulin resistance in *db/db* mice

**FIGURE 2:**
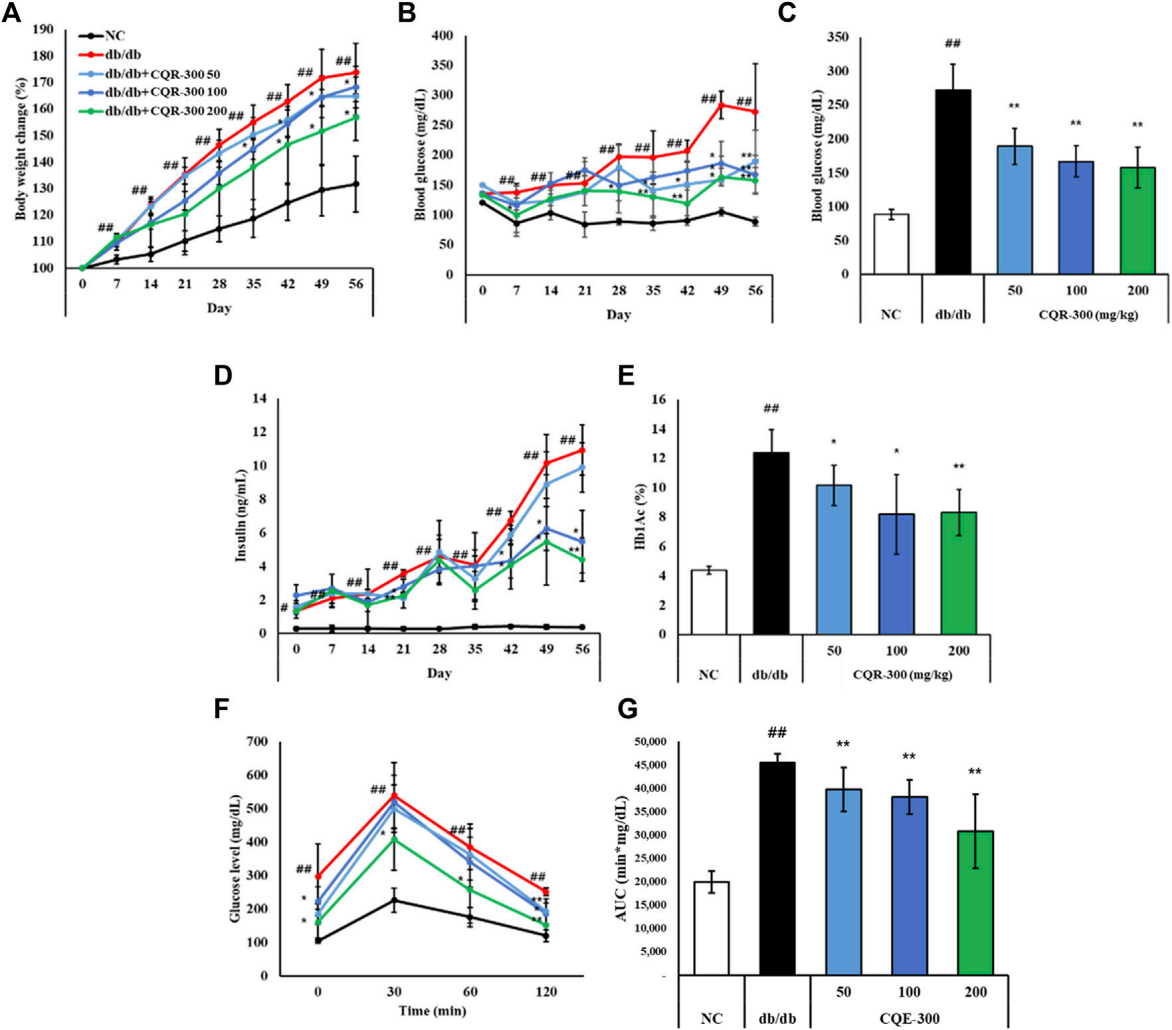
CQR-300 inhibits body weight increase and hyperglycemia. **(A)** Serial percent changes of body weight in 56 days. **(B)** Serial changes of glucose in 56 days. **(C)** Levels of glucose after 56 days of treatment with CQR-300. **(D)** Serial changes of insulin in 56 days. **(E)** HbA1c levels after 56 days of treatment with CQR-300. **(F)** Glucose profiles during oral glucose tolerance tests after 49 days of treatment with CQR-300. **(G)** Area under the curve of OGTT. Values are shown as means ± SD (n = 6). Significant differences: ^##^ *p* < 0.01 vs. NC; *,**p < 0.05 and *p* < 0.01 vs. *db/db*, respectively. CQR-300: *Cissus quadrangularis* extract; HbA1c: hemoglobin A1c.

Both *db/db* and NC mice showed increased body weights during the experiments. The percent change in body weight was significantly higher in the *db/db* group than in the NC group (Figure 2A, *p* < 0.01). However, after 42 days, the body weights of the CQR-300 100 mg/kg and CQR-300 200 mg/kg group were significantly lower than those of the *db/db* group (*p* < 0.05). There were no differences in the food and water intake of mice in any of the groups (Supplementary Figure S1). Fasting glucose and insulin levels were markedly higher in the *db/db* group than in the NC group (Figures 2B–D, *p* < 0.01). The difference between the *db/db*- and CQR-300-treated groups gradually increased over time. In all CQR-300-treated groups, fasting glucose and HbA1c levels were significantly reduced compared to those in the *db/db* group (Figures 2C–E). The recovery of glucose levels in the CQR-300-treated group was significantly faster than that in the *db/db* group (Figure 2F). Additionally, the area under the curve of OGTT in the CQE-300 treated group decreased in a dose-dependent manner compared to the *db/db* group (Figure 2G).

### 3.3 Effects of CQR-300 on insulin and glucagon expression in pancreas tissue of *db/db* T2DM mice

**FIGURE 3:**
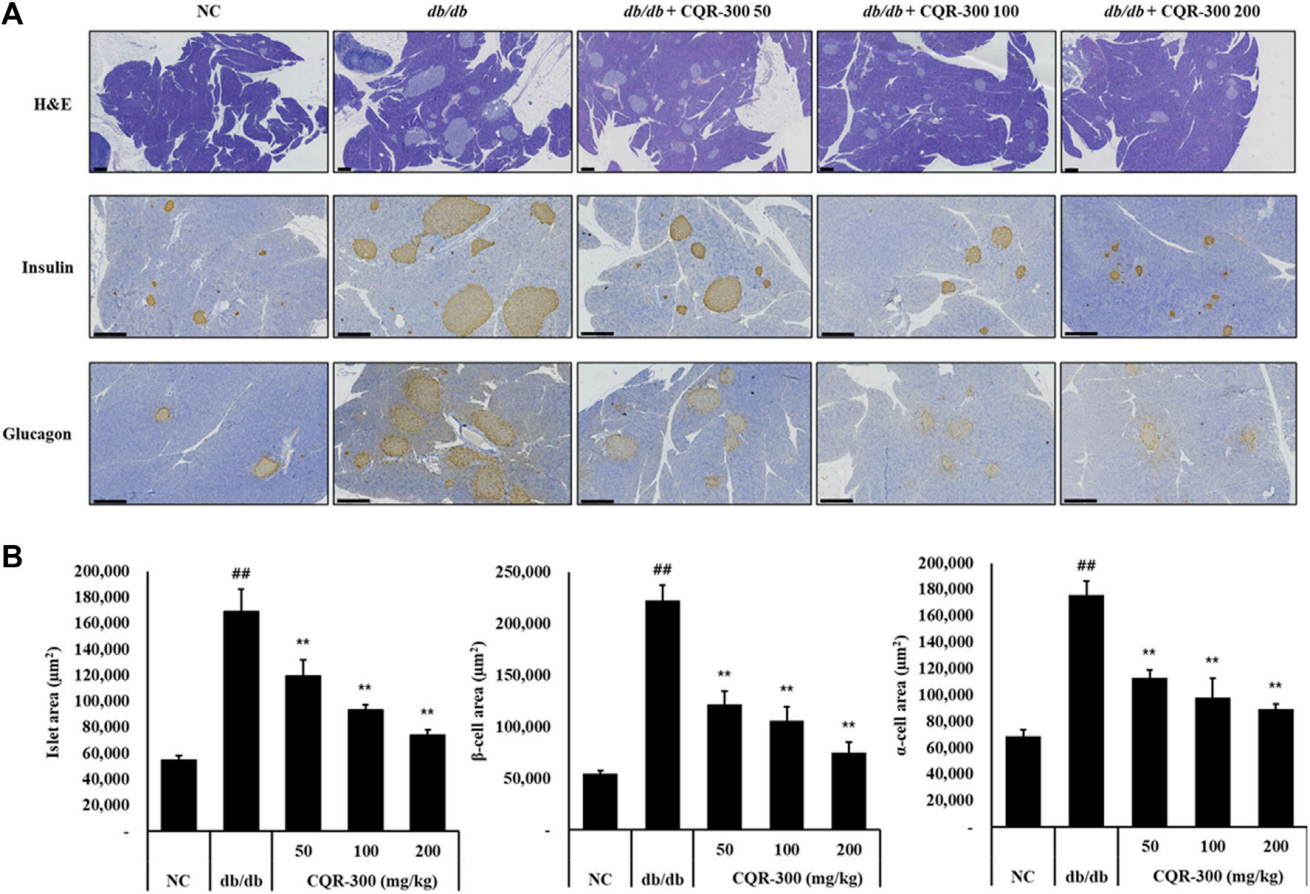
CQR-300 inhibits expression levels of insulin and glucagon in pancreatic tissue. **(A)** H&E staining and IHC analysis of insulin and glucagon in the pancreas. **(B)** Quantification of the islet, β-cell, and α-cell area in the pancreas. Values are shown as means ± SD (n = 6). Significant differences: ^##^ *p* < 0.01 vs. NC; *,**p < 0.05 and *p* < 0.01 vs. *db/db*, respectively. Bar scale = 300 µm. CQR-300: *C. quadrangularis* extract; H&E, hematoxylin and eosin; IHC, immunohistochemistry.

The islet area, β-cell area, and α-cell area in the pancreatic tissue were observed by H&E and IHC staining (Figure 3A). These areas were significantly larger for the *db/db* group than the NC groups (Figure 3B, *p* < 0.01). In all CQR-300-treated groups, these areas were significantly and dose-dependently downregulated (Figure 3B, *p* < 0.01).

### 3.4 Effect of CQR-300 on oxidative stress, lipogenesis in liver tissue of *db/db* mice

**FIGURE 4:**
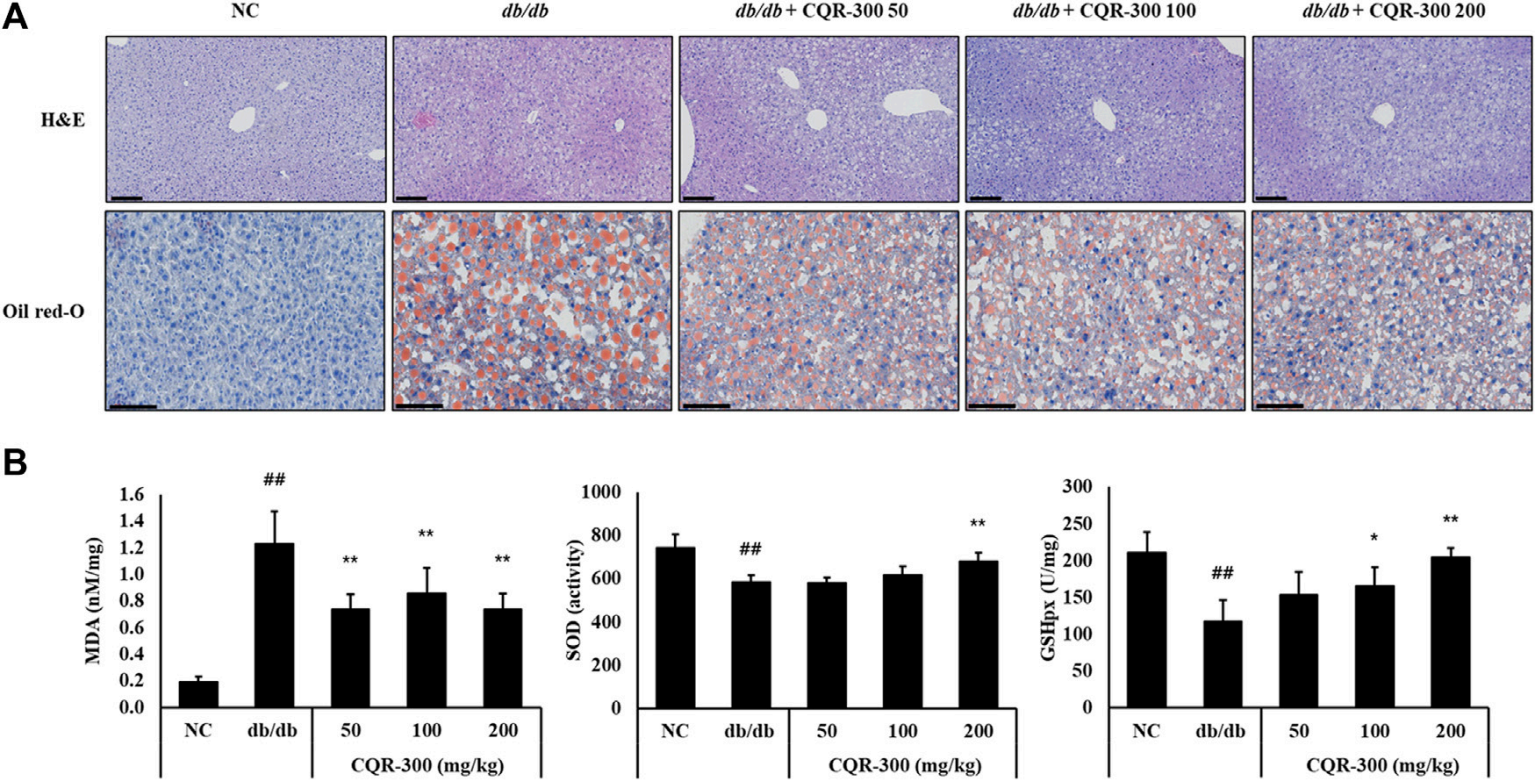
CQR-300 treatment inhibits the inflammation, lipid production, and oxidative stress in the liver. **(A)** H&E and Oil red-O staining of the liver. **(B)** Analysis of MDA, SOD, and GPX in the liver. Values are shown as means ± SD (n = 6). Significant differences: ^##^ *p* < 0.01 vs. NC; ^*,**^ *p* < 0.05 and *p* < 0.01 vs. *db/db*, respectively. Bar scale = 100 µm. CQR-300: *C. quadrangularis* extract; GPX, glutathione peroxidase; H&E, hematoxylin and eosin; MDA, malondialdehyde; SOD, superoxide dismutase.

As shown in Figure 4A, lipid accumulation in the liver tissue was observed by H&E and Oil red O staining in the *db/db* group. However, in the CQR-300-treated group, lipid accumulation decreased markedly. The clinical chemistry results (aspartate transaminase, alanine transferase, albumin, blood urea nitrogen, total cholesterol, high-density lipoprotein, creatinine, and uric acid) between the groups were consistent with normal variation (Supplementary Figure S2). In the *db/db* group, MDA levels were significantly increased, and SOD activity and GPX levels were significantly decreased (Figure 4B, *p* < 0.01). MDA levels in all CQR-300-treated groups were significantly reduced (*p* < 0.01), and SOD activity and GPX levels in the CQR-300 treated groups were dose-dependently upregulated.

### 3.5 Effects of CQR-300 on gluconeogenesis, lipogenesis, and oxidative stress in liver tissue of *db/db* mice

**FIGURE 5:**
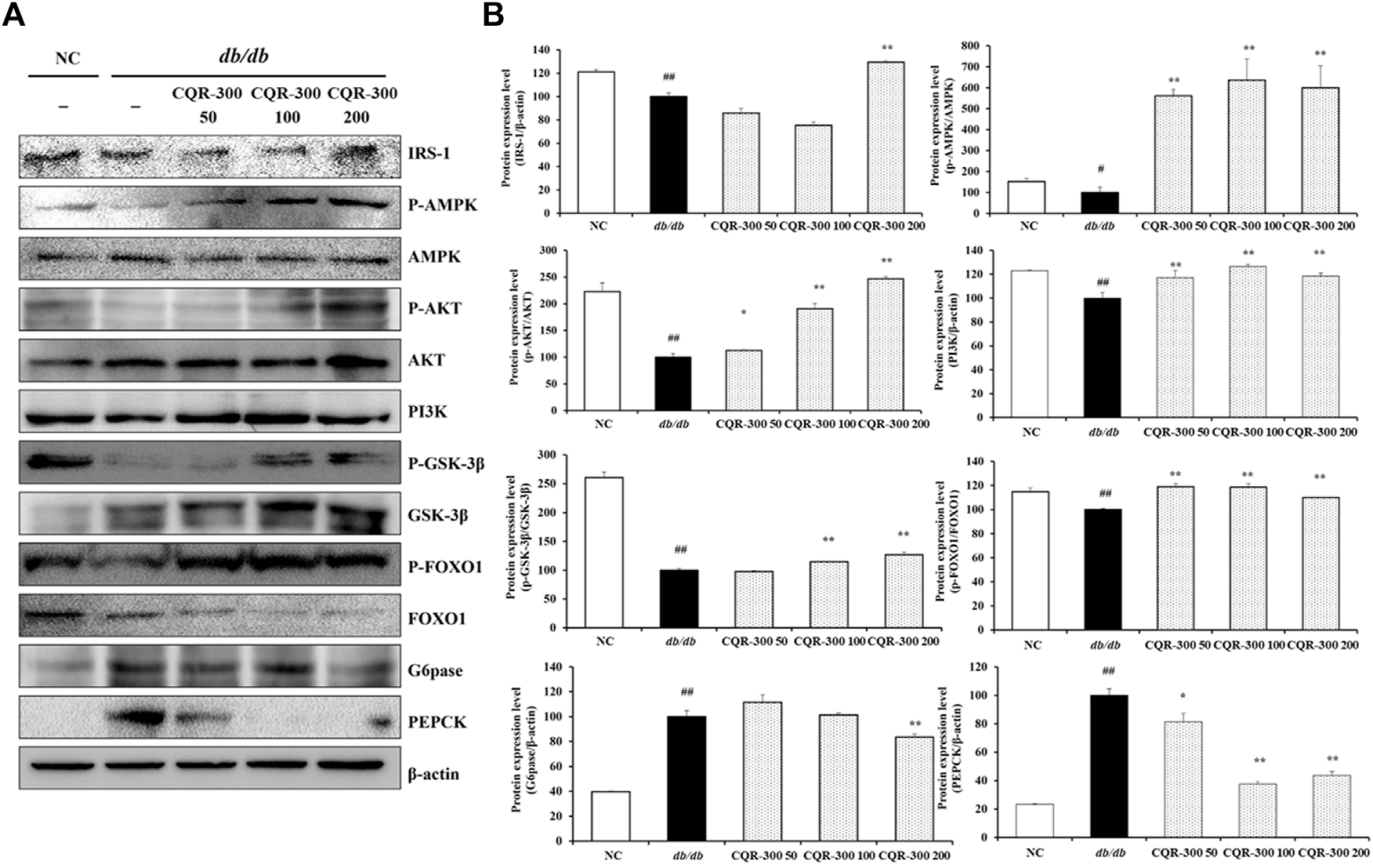
CQR-300 reduces gluconeogenesis in liver tissue. **(A)** Western blot analysis of gluconeogenesis-related factors in the liver. **(B)** Densitometric values were calculated using ChemiDoc. Values are shown as means ± SD (n = 6). Significant differences: ^##^ *p* < 0.01 vs. NC; ^*,**^ *p* < 0.05 and *p* < 0.01 vs. *db/db*, respectively. CQR-300: *C. quadrangularis* extract.

**FIGURE 6:**
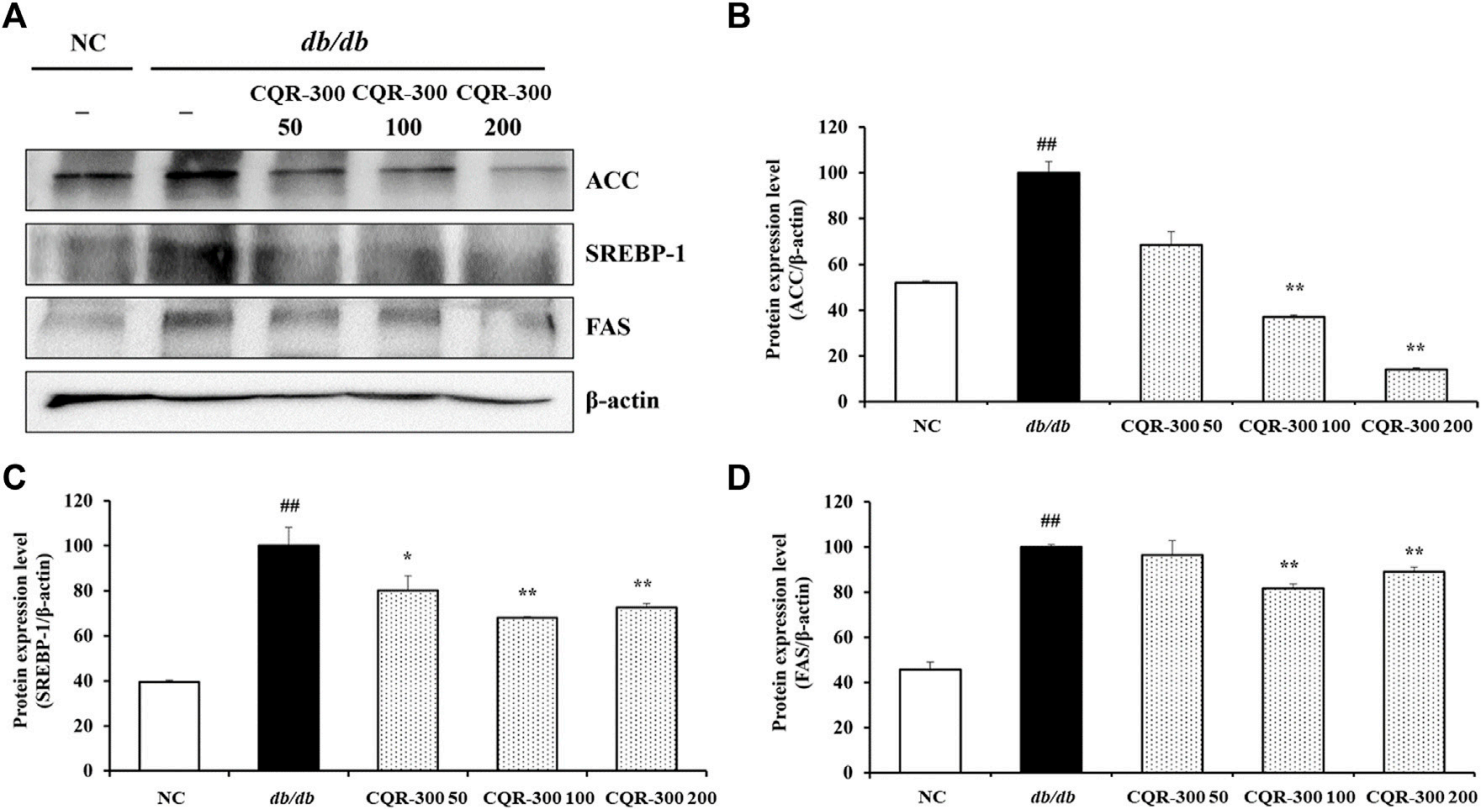
CQR-300 reduces lipogenesis in liver tissue. **(A)** Western blot analysis of lipogenesis-related factors in the liver. **(B–D)** Densitometric values were calculated using ChemiDoc. Values are shown as means ± SD (n = 6). Significant differences: ^##^ *p* < 0.01 vs. NC; ^*,**^ *p* < 0.05 and *p* < 0.01 vs. *db/db*, respectively. CQR-300: *C. quadrangularis* extract.

**FIGURE 7:**
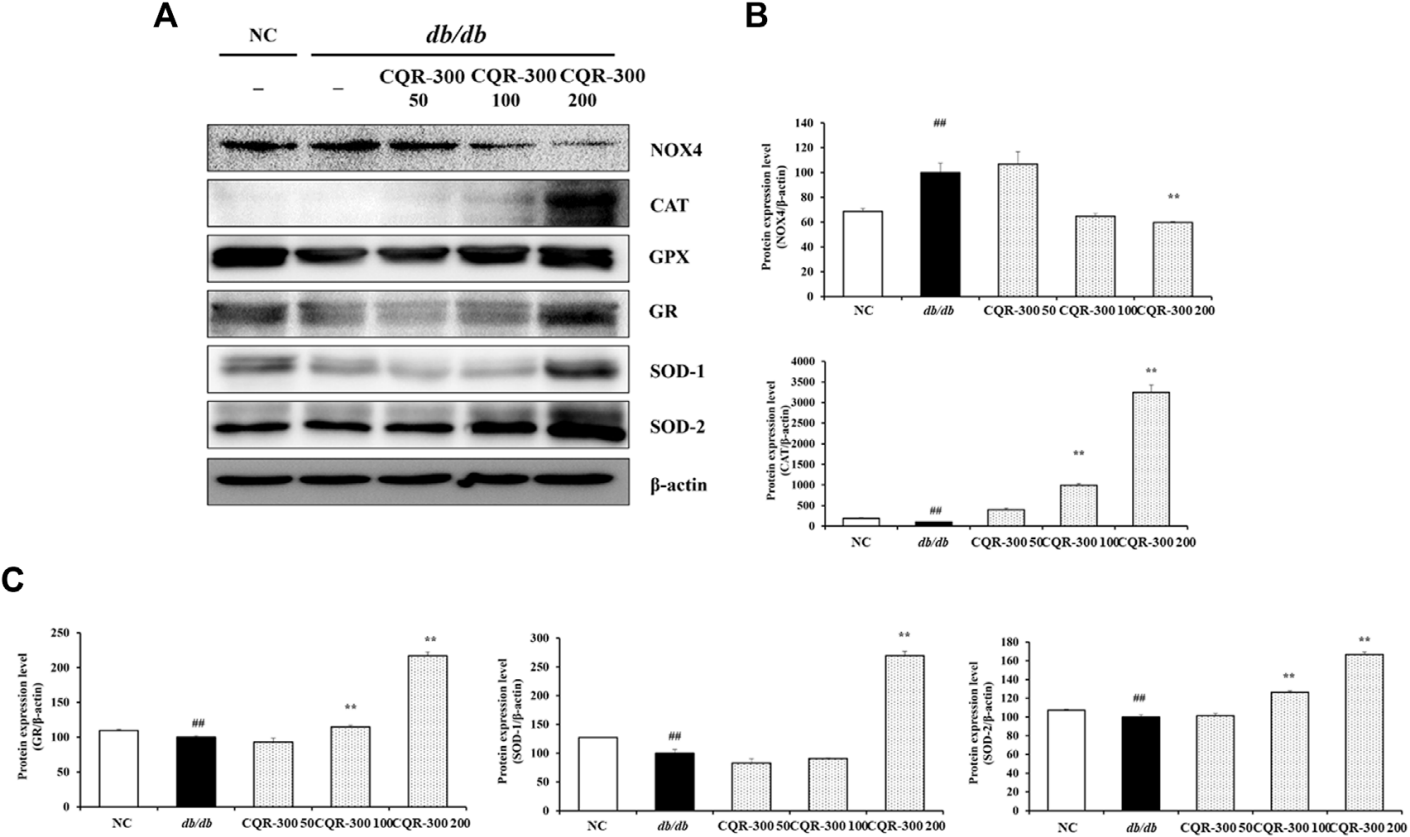
CQR-300 reduces oxidative stress in liver tissue. **(A)** Western blot analysis of oxidative stress-related factors in the liver. **(B,C)** Densitometric values were calculated using ChemiDoc. Values are shown as means ± SD (*n* = 6). Significant differences: ^##^ *p* < 0.01 vs. NC; ^*,**^ *p* < 0.05 and *p* < 0.01 vs. *db/db*, respectively. CQR-300: *C. quadrangularis* extract.

Western blotting of the liver tissues was performed to evaluate the levels of gluconeogenesis, lipogenesis, and oxidative stress-related factors (Figures 5–7). The *db/db* group showed significantly lower levels of gluconeogenesis-related factors, such as IRS-1 and PI3K, than the NC group (Figure 5). Additionally, the phosphorylation of AMPK, AKT, GSK-3β, and FOXO1 was significantly decreased in the *db/db* group than NC group. Contrarily, the expression of G6pase and PEPCK in the *db/db* group was markedly higher than those in the NC group (*p* < 0.01). CQR-300-treated groups, especially in the CQR-300 200 mg/kg group (*p* < 0.01), had an upregulated expression of IRS-1 and PI3K and phosphorylation of AMPK, AKT, GSK-3β, and FOXO1 compared to the *db/db* group. Additionally, in the CQR-300 200 mg/kg group, the expression levels of G6pase and PEPCK were downregulated compared to those in the *db/db* group (*p* < 0.01).

The *db/db* group showed significantly higher expression levels of lipogenesis-related factors, such as ACC, SREBP-1, and FAS, than the NC group (Figure 6, *p* < 0.01). Conversely, CQR-300-treated groups, especially the CQR-300 100 mg/kg and CQR-300 200 mg/kg groups (*p* < 0.01), showed downregulated expression of ACC, SREBP-1, and FAS compared to the *db/db* group.

The levels of oxidative stress-related factors, such as CAT, GPX, GR, SOD-1, and SOD-2, were significantly lower in the *db/db* group than the NC group (Figure 7). The *db/db* group exhibited significantly higher NOX4 levels than the NC group. In contrast, the expression of CAT, GPX, GR, SOD-1, and SOD-2 in the CQR-300-treated groups was upregulated in a dose-dependent manner compared with that in the *db/db* group, especially in the CQR-300 200 mg/kg group (*p* < 0.01). The CQR-300-treated groups, especially the CQR-300 200 mg/kg group (*p* < 0.01), showed downregulated expression of NOX4 compared with the *db/db* group.

## 4 Discussion

Due to mutations in the leptin receptor, *db/db* mice exhibit elevated blood insulin levels and hyperglycemia, making them a common choice for diabetes research (Rőszer et al., 2014). The present study aimed to assess the potential therapeutic effect of CQR-300 in treating T2DM in *db/db* mice by focusing on its effects on gluconeogenesis, adipogenesis, and oxidative stress.

Moderate weight loss provides significant benefits to patients with obesity and T2DM (Kahan and Fujioka, 2017). Weight loss in patients with diabetes is challenging as they consistently lose less weight with a given treatment than those who do not have diabetes. This is particularly notable in obesity pharmacotherapy trials, in which weight loss is generally 25% lower in patients with obesity and diabetes than in patients with obesity without diabetes. In this study, despite no differences in food and water intake between the groups, an 8-week oral administration of CQR-300 effectively suppressed weight gain in *db/db* mice and reduced the levels of HbA1c, insulin, and fasting blood glucose, which serve as markers for insulin resistance.

The underlying cause of hypoglycemia in diabetes is a complex interaction between hyperinsulinemia and compromised physiological and behavioral responses to reduced glucose levels. Clinical studies in patients with diabetes receiving anti-obesity medications have reported a risk of hypoglycemia in some patients (Oyer, 2013). Contrary to concerns about hypoglycemic symptoms due to weight loss induced by CQR-300, the fasting blood glucose levels in the CQR-300-treated group were within the normal range. Additionally, CQR-300-treated mice exhibited reduced glucose spikes during OGTTs, leading to a faster return to baseline glucose levels than in the *db/db* group.

Insulin resistance, a major contributing factor to T2DM, is defined as a physiological state in which tissues are insensitive to the action of insulin (Pei et al., 2022). Although the cause-and-effect relationship between insulin resistance and hyperinsulinemia is still debated, insulin resistance is considered the primary factor, and compensatory hyperinsulinemia ensues during T2DM progression (Abdul-Ghani and DeFronzo, 2021). In the present histological analysis, CQR-300 treatment decreased the islet diameter and reduced insulin and glucagon secretion in pancreatic tissue compared to that in the *db/db* group. Considering the lower blood glucose levels and β-cell size, along with the corresponding reduced insulin levels in the current CQR-300-treated group, it is speculated that CQR-300 may inhibit gluconeogenesis and prevent the worsening of insulin resistance in T2DM by reducing glucose-stimulated insulin secretion.

Western blotting was performed to analyze the expression levels of key factors involved in gluconeogenesis, glycolysis, and the PI3K/Akt pathway in the liver. These results provided additional evidence that CQR-300 treatment inhibited gluconeogenesis and promoted glycogen synthesis, thereby reducing glucose levels in the liver. The IRS-1/PI3K/AKT signaling pathway is known to reduce hepatic glucose production and glycogenolysis by enhancing FOXO1 and GSK-3β activity (Titchenell et al., 2016). We found that both PI3K and AKT were suppressed in *db/db* mice, which is consistent with the results of previous studies (Bai et al., 2020). However, 200 mg/kg treatment of CQR-300 led to increased expression of the IRS-1/PI3K/AKT pathway and increased phosphorylation of GSK-3β and FOXO1, which was followed by decreased expression levels of G6pase and PEPCK, when compared to the *db/db* group. Previous studies have confirmed that leptin has the ability to either hinder gluconeogenesis or amplify insulin’s inhibitory impact on glycogenolysis (Hussain and Khan, 2017). Moreover, leptin reported to involve in blood glucagon reduction, which is an enzyme for gluconeogenesis, and to promote glycogen storage in hepatocytes by inhibiting glycogen phosphorylase and GSK-3β (Rossetti et al., 1997; Denroche et al., 2012). The present restored phosphorylation of PI3K, AKT, and IRS-1, which are the downstream signals of the leptin receptor after CQE-300 administration, suggesting that CQE-300 partially regulated the gluconeogenesis under leptin receptor mutated condition.

Glucose and lipid metabolism are intricately interconnected in various aspects (Dong et al., 2023). A recent meta-analysis of the association between nonalcoholic fatty liver disease (NAFLD) and T2DM found that NAFLD doubled the risk of diabetes (Mantovani et al., 2018). Under NAFLD conditions, the enhanced expression of proteins associated with lipogenesis-related genes, including ACC, SREBP-1, and FAS, in hepatocytes leads to increased insulin resistance (Hsu et al., 2016). Numerous reports have demonstrated that SREBP-1 induces lipogenic enzymes, contributing to the lipid deposition associated with insulin resistance (Moslehi and Hamidi-Zad, 2018). In this study, CQR-300 treatment successfully suppressed ACC, SREBP-1, and FAS signals by enhancing AMPK phosphorylation. The histological analysis of liver tissue with Oil red-O-staining further supported the anti-lipogenic properties of CQR-300, revealing a reduced fat accumulation in CQR-300 treated groups compared to the *db/db* alone group. This was also in line with a previous study that found reduced white adipose tissue due to enhanced AMPK phosphorylation in CQR-300-treated high-fat diet mice (Lee et al., 2016). The observed reduction in lipid accumulation following CQR-300 treatment may be, at least in part, a contributing factor to improved insulin resistance in *db/db* mice. Additionally, CQR-300 treatment improved oxidative stress by enhancing the activity of antioxidant enzymes such as SOD, GPX, and CAT; upregulating GR, SOD-1, and SOD-2; and suppressing the source of oxidative stress, NOX-4. Polyphenolic compounds including quercetin, resveratrol, catechin, kaempferol contained in CQR-300 may be responsible for significantly relieving oxidative stress in the liver of *db/db* mice (Avula et al., 2021). Quercetin could increase GSH, SOD, and CAT activity, lower blood glucose and insulin levels, and reduce the lipid accumulation of the liver in *db/db* mice (Jeong et al., 2012; Yang et al., 2019). These studies strongly support our results.

This study has several limitations that make it difficult to translate to humans. Although *db/db* mice are suitable for obesity and T2DM research, leptin mutations result in extreme obesity and diabetes symptoms, leading to significant phenotypic changes that make it challenging to understand the progression stages of human diseases (Fang et al., 2010). Moreover, the present highest dose (200 mg/kg) in mice was 972 mg/60 kg based on human equivalent dose, which was lower than the human average daily intake for *C. quadrangularis* powder is 1,000–1,400 mg/60 kg as a human health functional food. Although there are metabolic differences between species, it would have been better to set a higher dose than the current one to mimic the human daily intake.

## 5 Conclusion

To the best of our knowledge, this is the first study which shows that CQR-300 provides protective effects in *db/db* mice, primarily by regulating gluconeogenesis, adipogenesis, and oxidative stress, which may improve insulin resistance in T2DM progression. These findings provide a scientific basis for the application of CQR-300 as a natural anti-diabetic drug or functional food because of its multitarget anti-hyperglycemic effect. Further studies are needed to explore the effects of CQR-300 on gluconeogenesis in various disease models.

## Data Availability

The raw data supporting the conclusions of this article will be made available by the authors, without undue reservation.
