# Supplementary material for: Anti-hyperglycemic effects of Cissus quadrangularis extract via regulation of gluconeogenesis in type 2 diabetic db/db mice
Source: Front Pharmacol. 2024 Jul 10;15:1415670. doi: 10.3389/fphar.2024.1415670 (PMC11266303; doi:10.3389/fphar.2024.1415670)
Supplement: Supplementary file 1 [file Table1.DOCX]

Supplementary Material

# TABLE S1.

| **Antibodies** | **Distributor** | **Dilutions** |
| --- | --- | --- |
| P-AMPK | Cell Signaling Technology (Danvers, MA, USA) | 1:1000 |
| AMPK | Cell Signaling Technology |  |
| ACC | Cell Signaling Technology |  |
| FAS | Cell Signaling Technology |  |
| IRS-1 | Cell Signaling Technology |  |
| P-AKT | Cell Signaling Technology |  |
| AKT | Cell Signaling Technology |  |
| PI3K | Cell Signaling Technology |  |
| P-GSK-3β | Cell Signaling Technology |  |
| GSK-3β | Cell Signaling Technology |  |
| P-FOXO1 | Cell Signaling Technology |  |
| FOXO1 | Cell Signaling Technology |  |
| GPX | Santa Cruz Biotechnology  (Santa Cruz, CA, USA) | 1:500 |
| SOD-1 | Santa Cruz Biotechnology |  |
| SOD-2 | Santa Cruz Biotechnology |  |
| GR | Santa Cruz Biotechnology |  |
| NOX4 | Santa Cruz Biotechnology |  |
| SREBP-1 | Santa Cruz Biotechnology |  |
| PEPCK | Santa Cruz Biotechnology |  |
| G6pase | Abcam (Cambridge, UK) | 1:1000 |
| Catalase | Cell Signaling Technology |  |
| β-actin | Cell Signaling Technology |  |
| Horseradish peroxidase (HRP)–conjugated anti-rabbit IgG, HRP-conjugated anti-mouse IgG | GenDEPOT (Barker, TX, USA) | 1:10000 |
