# Supplementary material for: Anti-hyperglycemic effects of Cissus quadrangularis extract via regulation of gluconeogenesis in type 2 diabetic db/db mice
Source: Front Pharmacol. 2024 Jul 10;15:1415670. doi: 10.3389/fphar.2024.1415670 (PMC11266303; doi:10.3389/fphar.2024.1415670)
Supplement: Supplementary file 2 [file Presentation1.PPTX]

## Slide 1
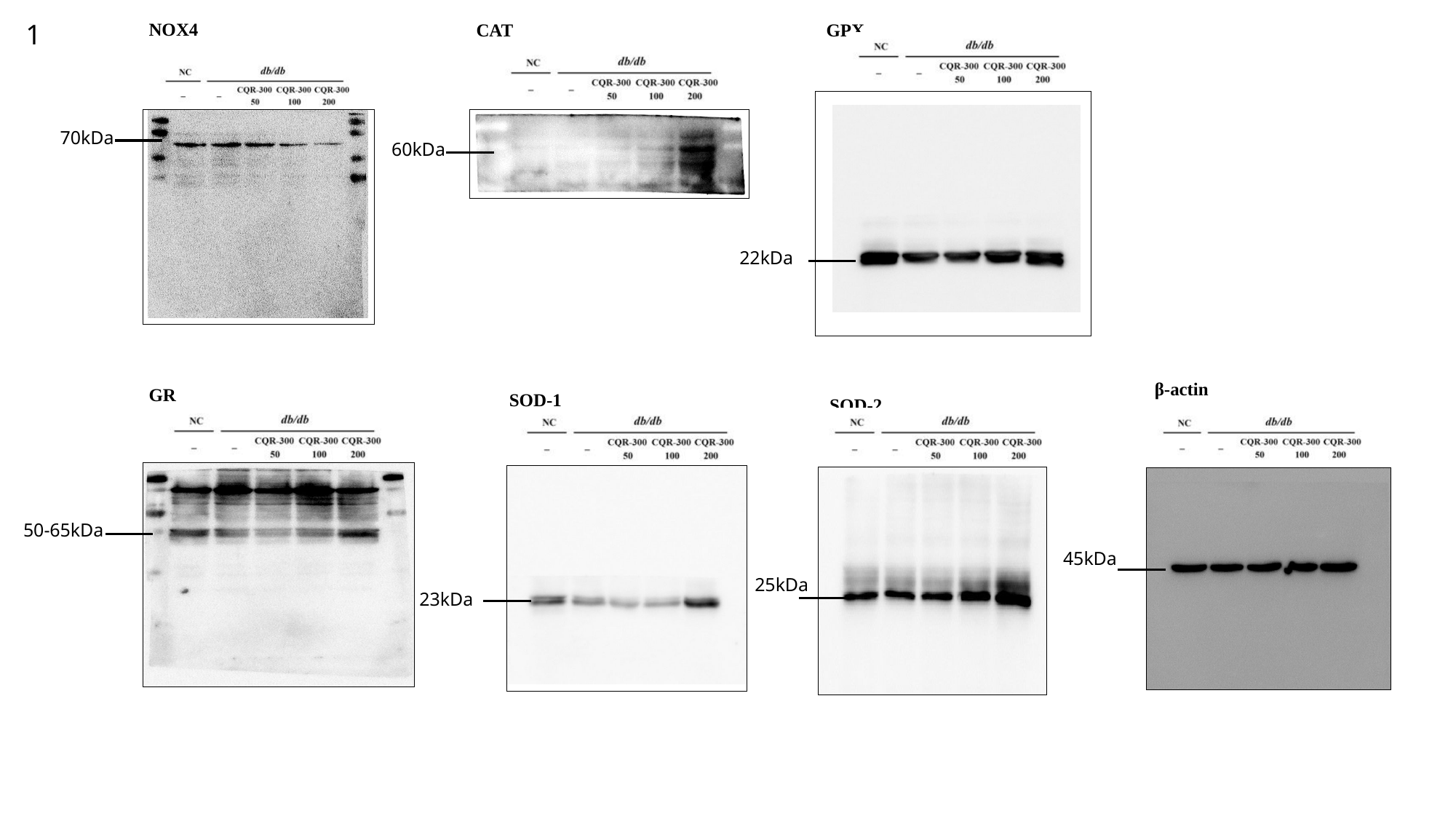

1
NOX4
CAT
GPX
70kDa
60kDa
22kDa
β-actin
GR
SOD-1
SOD-2
50-65kDa
45kDa
25kDa
23kDa

## Slide 2
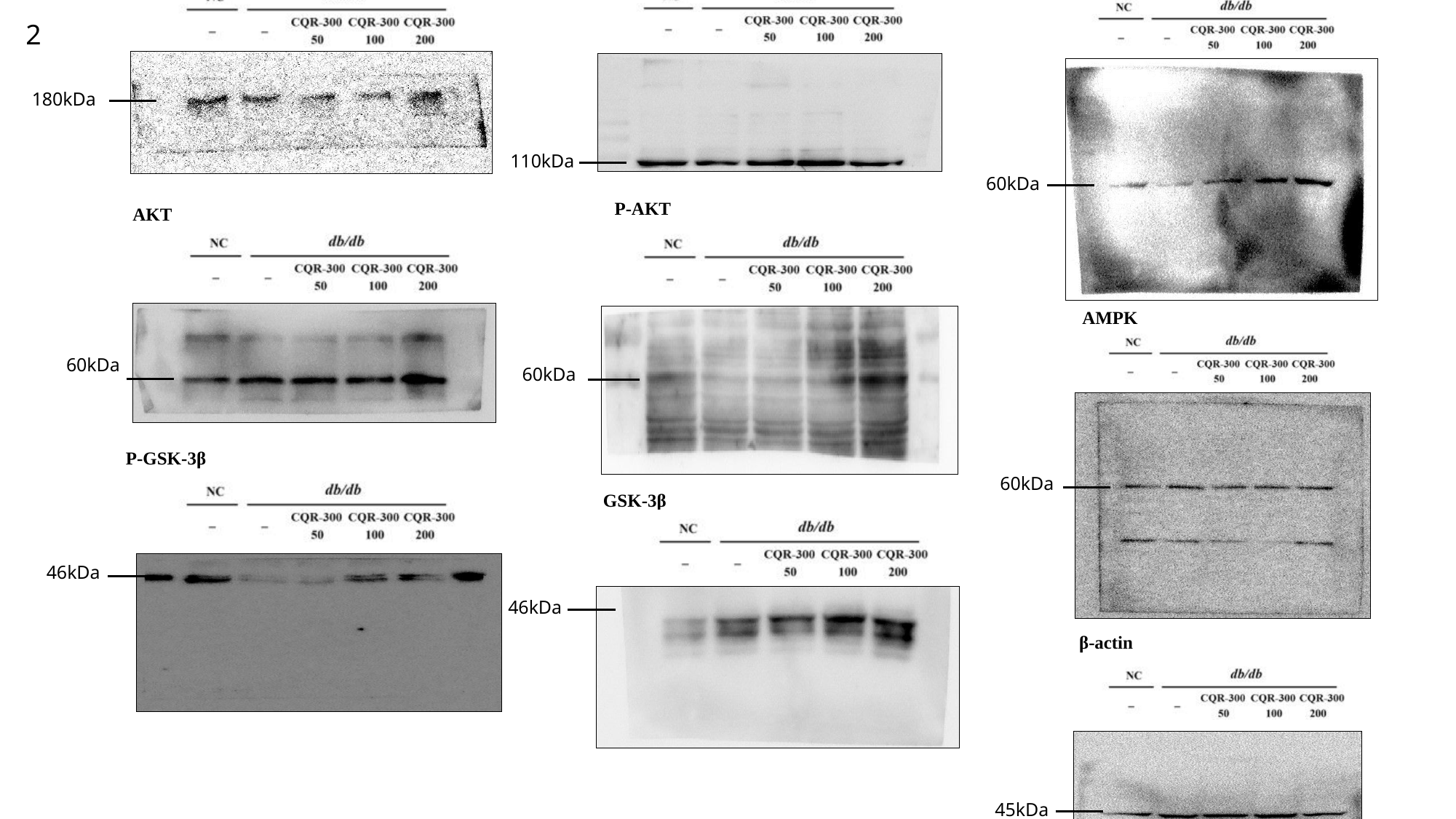

PI3K
IRS-1
P-AMPK
2
180kDa
110kDa
60kDa
P-AKT
AKT
AMPK
60kDa
60kDa
P-GSK-3β
60kDa
GSK-3β
46kDa
46kDa
β-actin
45kDa

## Slide 3
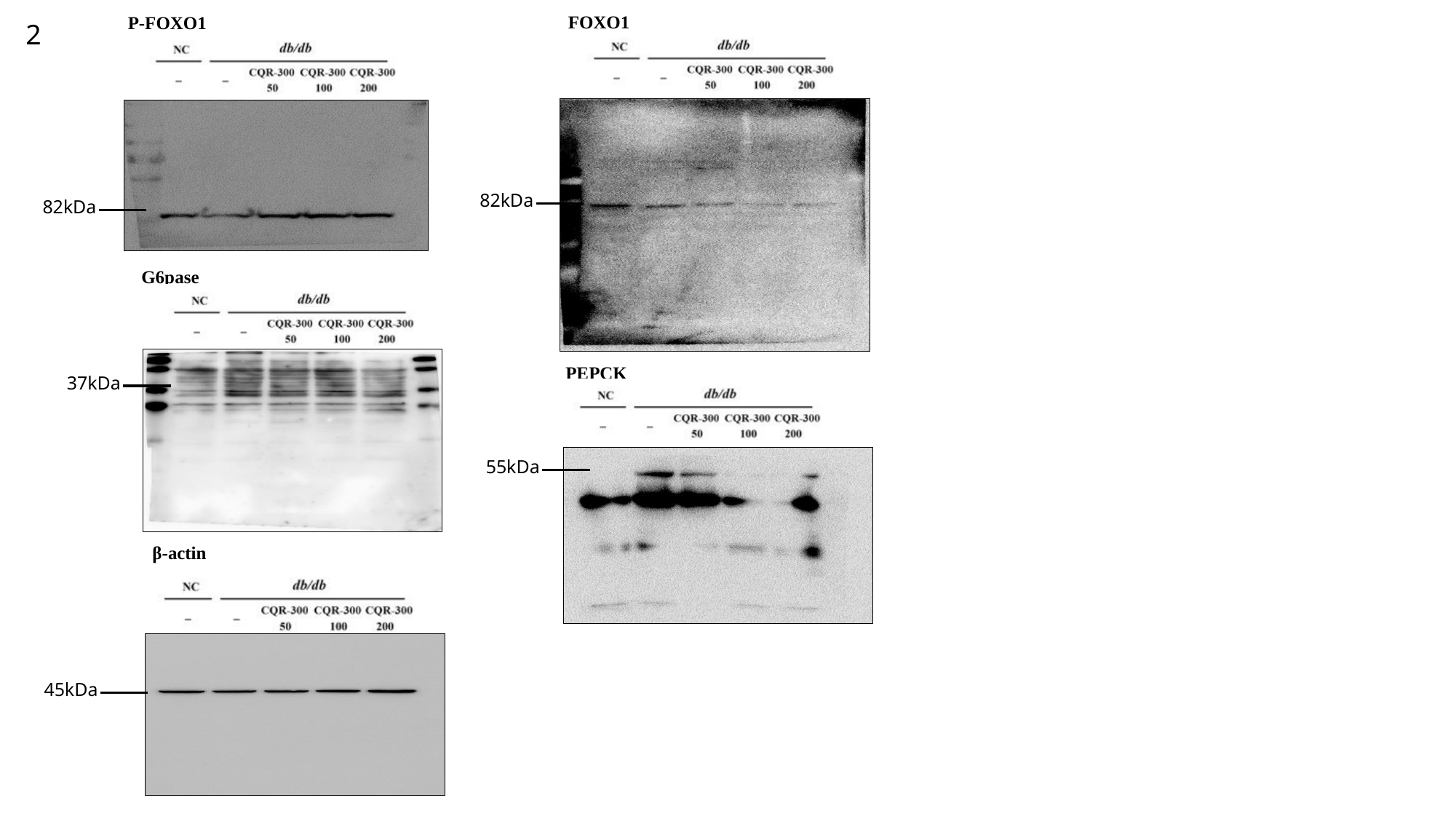

FOXO1
P-FOXO1
2
82kDa
82kDa
G6pase
PEPCK
37kDa
55kDa
β-actin
45kDa

## Slide 4
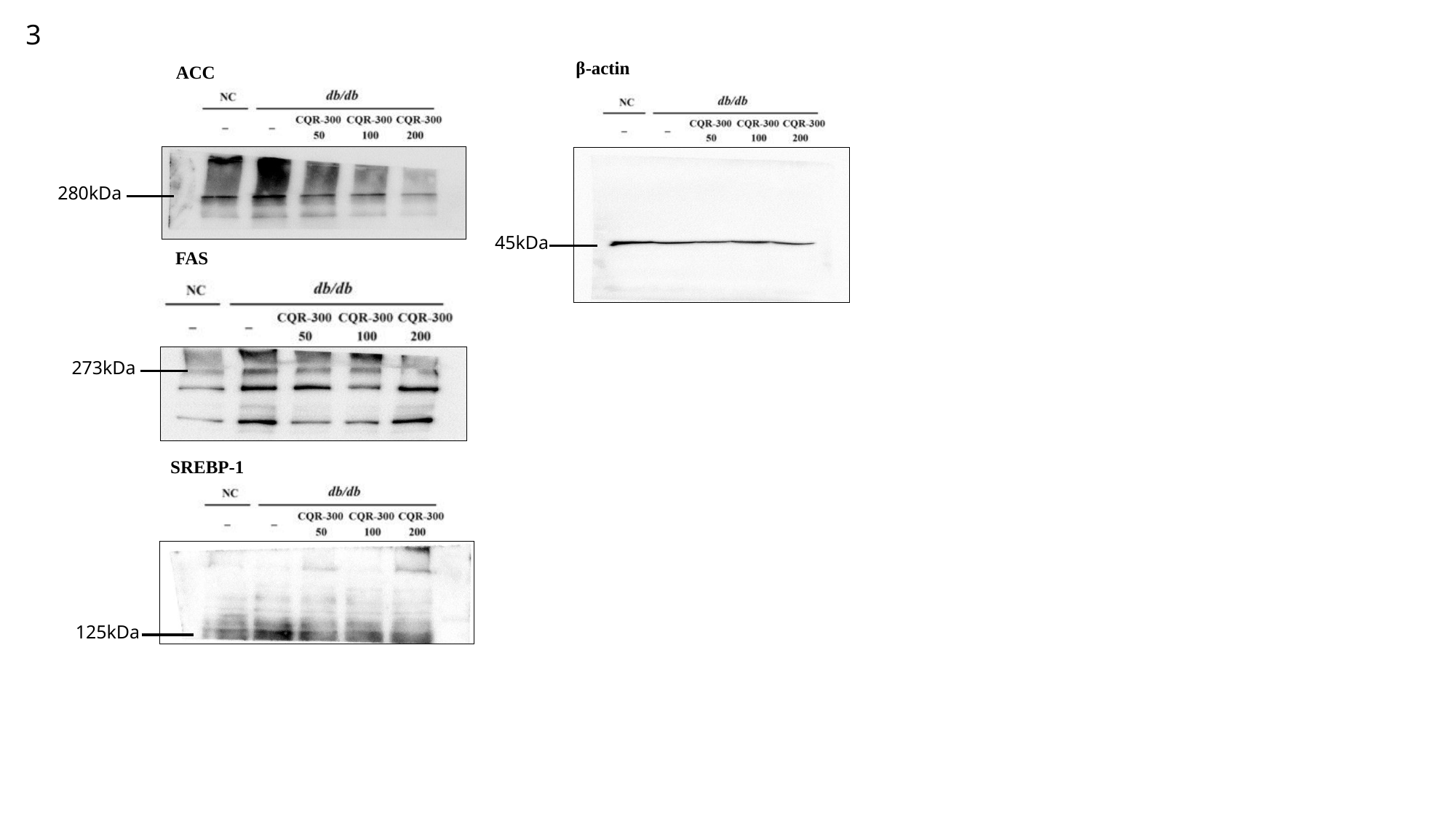

3
β-actin
ACC
280kDa
45kDa
FAS
273kDa
SREBP-1
125kDa
