# Supplementary figures and images for: Anti-hyperglycemic effects of Cissus quadrangularis extract via regulation of gluconeogenesis in type 2 diabetic db/db mice
Source: Front Pharmacol. 2024 Jul 10;15:1415670. doi: 10.3389/fphar.2024.1415670 (PMC11266303; doi:10.3389/fphar.2024.1415670)

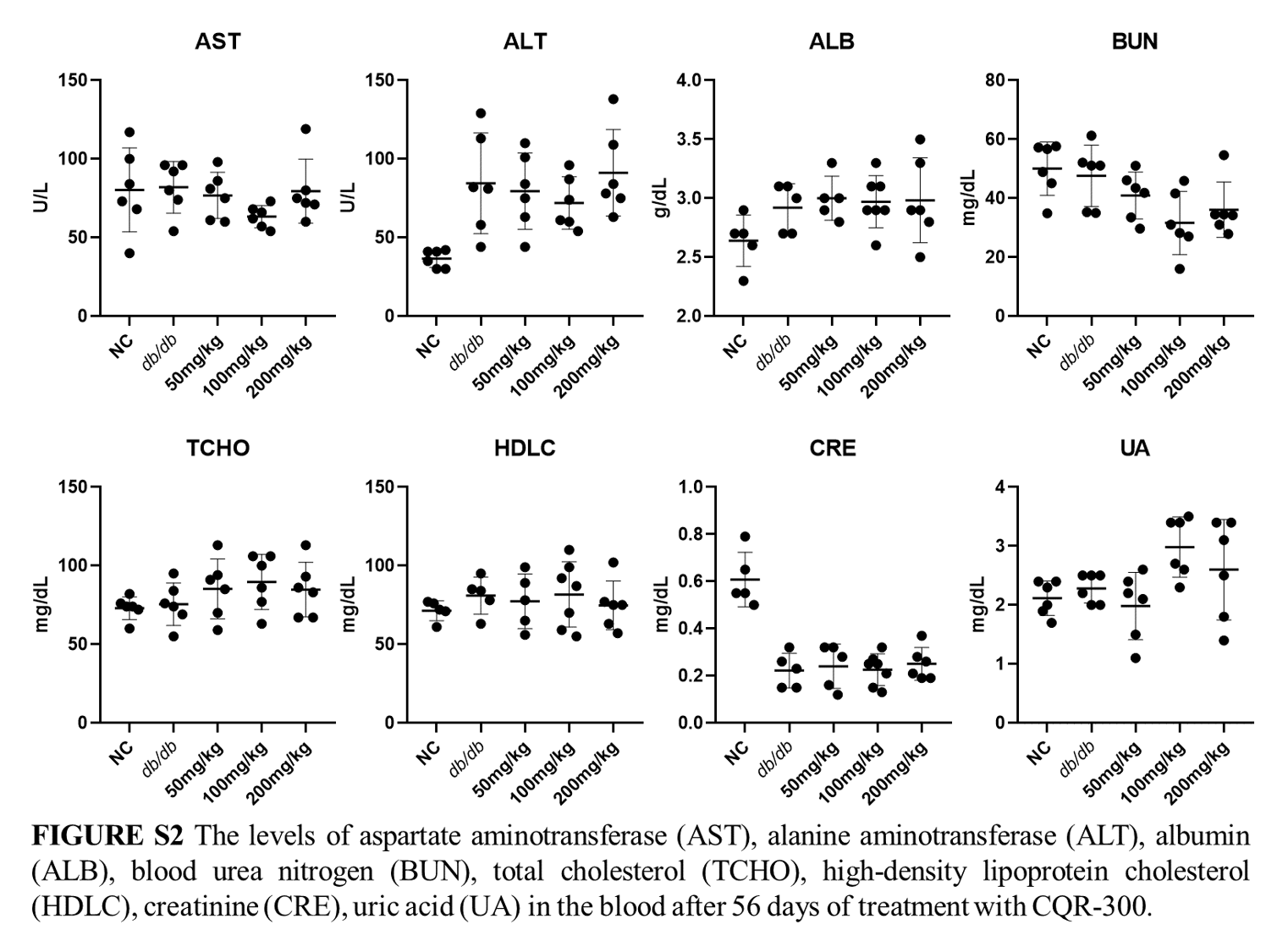

Supplement: Supplementary file 3 [file Image2.TIF]

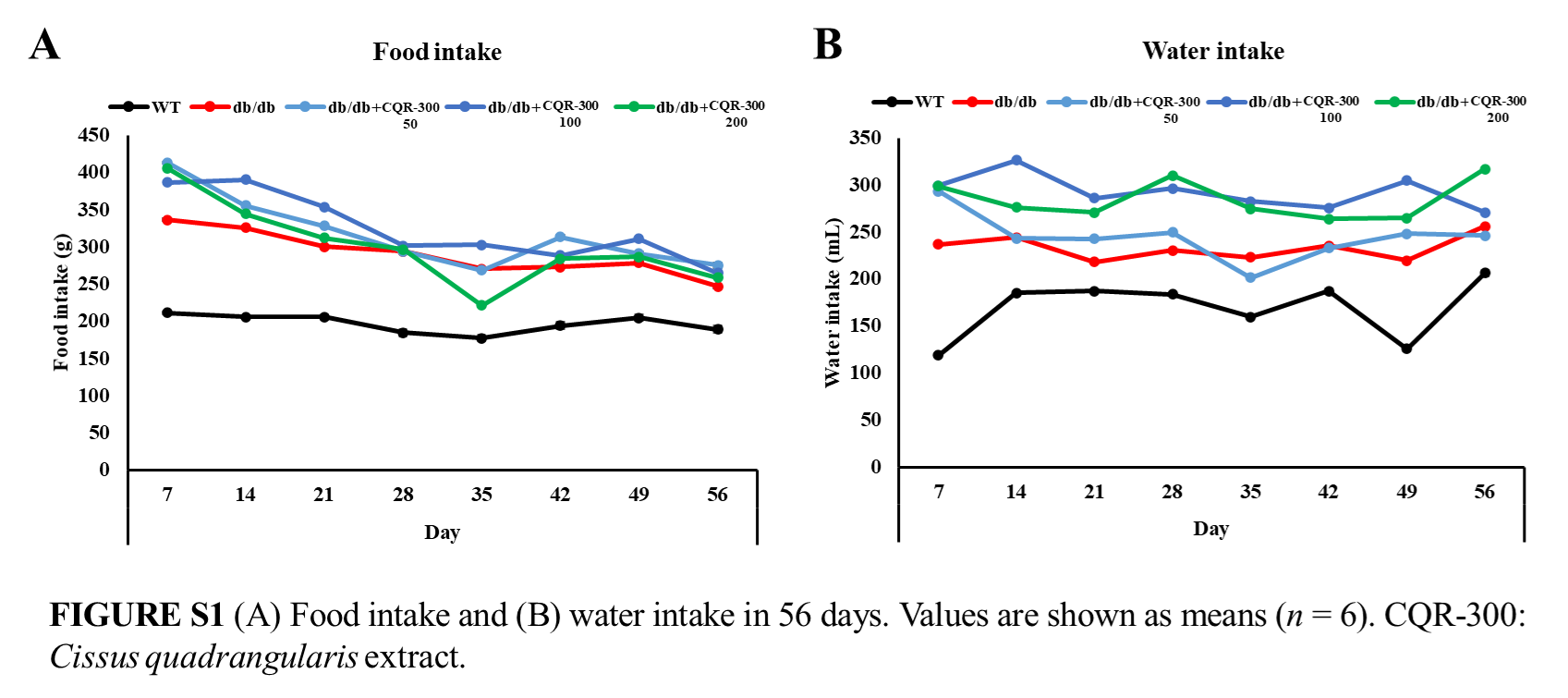

Supplement: Supplementary file 4 [file Image1.TIF]
